# Phylogenomic analysis of the GIY-YIG nuclease superfamily

**DOI:** 10.1186/1471-2164-7-98

**Published:** 2006-04-28

**Authors:** Stanislaw Dunin-Horkawicz, Marcin Feder, Janusz M Bujnicki

**Affiliations:** 1 Laboratory of Bioinformatics and Protein Engineering, International Institute of Molecular and Cell Biology, Trojdena 4, 02-109 Warsaw, Poland

## Abstract

**Background:**

The GIY-YIG domain was initially identified in homing endonucleases and later in other selfish mobile genetic elements (including restriction enzymes and non-LTR retrotransposons) and in enzymes involved in DNA repair and recombination. However, to date no systematic search for novel members of the GIY-YIG superfamily or comparative analysis of these enzymes has been reported.

**Results:**

We carried out database searches to identify all members of known GIY-YIG nuclease families. Multiple sequence alignments together with predicted secondary structures of identified families were represented as Hidden Markov Models (HMM) and compared by the HHsearch method to the uncharacterized protein families gathered in the COG, KOG, and PFAM databases. This analysis allowed for extending the GIY-YIG superfamily to include members of COG3680 and a number of proteins not classified in COGs and to predict that these proteins may function as nucleases, potentially involved in DNA recombination and/or repair. Finally, all old and new members of the GIY-YIG superfamily were compared and analyzed to infer the phylogenetic tree.

**Conclusion:**

An evolutionary classification of the GIY-YIG superfamily is presented for the very first time, along with the structural annotation of all (sub)families. It provides a comprehensive picture of sequence-structure-function relationships in this superfamily of nucleases, which will help to design experiments to study the mechanism of action of known members (especially the uncharacterized ones) and will facilitate the prediction of function for the newly discovered ones.

## Background

The GIY-YIG superfamily groups together nucleases characterized by the presence of a domain of typically ~100 aa, with two short motifs "GIY" and "YIG" in the N-terminal part, followed by an Arg residue in the center and a Glu residue in the C-terminal part [1]. The GIY-YIG domain has been originally identified in a group of homing endonucleases (HEases). 'Homing' is a gene conversion process that occurs in Eukaryota, Archaea, Bacteria, and viruses, where a mobile sequence (a group I, group II, or archaeal intron or an intein) is copied and inserted into a cognate allele. It is initiated by a double-strand cut in the target allele, catalyzed by a HEase encoded within the mobile element (reviews: [2,3]). Unlike transposases, HEases do not recognize their mobile DNA, only recognize and cleave the DNA that possesses a non-interrupted target site. HEases are polyphyletic and belong to at least three structurally unrelated superfamilies of nucleases: GIY-YIG, ββα Me (including HNH and His-Cys box families), and LAGLIDADG (review: [4]).

Despite completely different structures and modes of interaction with the target DNA, they are all characterized by an extended binding site, conferred by long loops or additional domains, which allows them to recognize extremely long targets (even > 40bp). HEases, however, do not have stringently-defined recognition sequences and they usually tolerate single or even multiple base changes, which allows them for invading different alleles in the same genome and perhaps in other genomes [4]. The structure of I-TevI HEase was determined in two parts by X-ray crystallography. The C-terminal DNA-binding domain exhibits an unusually extended structure containing a Zn-finger, a minor groove-binding α-helix and a helix-turn-helix motif [5]. The N-terminal GIY-YIG domain was found to exhibit a unique three-dimensional fold comprising three β-strands surrounded by three α-helices [6], in a good agreement with our earlier prediction [7].

To date, members of the GIY-YIG superfamily have been found only in group I introns, and not in group II or archaeal introns or in inteins. However, they have been also identified as free-standing open reading frames (ORFs) in Bacteria and viruses [1]. Nucleases of the Seg family that are encoded in intragenic regions of T4 phage act in a similar way to their intron-encoded relatives. In mixed infections with the related phage T2 that lacks *seg *genes, they mediate "intronless homing", resulting in replacement of non-homologus T2 DNA with their self DNA [8]. On the other hand, endonuclease II of phage T4 (Endo II), another member of the GIY-YIG superfamily, is used by the phage to degrade the bacterial DNA, which allows reutilization of the bases for synthesis of the phage DNA [9]. Normal T4 DNA is protected from degradation by modification (hydroxymethylation and glucosylation) of cytosine residues. This process is very similar to the *modus operandi *of restrictrion-modification (RM) systems, which comprise a restriction endonuclease (REase) that degrades foreign DNA by cleaving specific target sites and a methyltransferase (MTase) that modifies bases in the targets in the self DNA to render them resistant to cleavage (reviews: [10-12]). The amino acid sequences of REases are extremely diverse, which makes them very difficult targets for phylogenetic classification [13]. Those REases, whose structures have been determined by crystallography, were found to belong to the PD-(D/E)XK superfamily of nucleases, completely unrelated to all superfamilies of HEases. However, we have recently found a small subgroup comprising just three closely related REases which belong to the GIY-YIG superfamily [14]. The GIY-YIG nuclease domain has been also found in a large protein encoded by *Penelope*-like non-LTR retroelements that also includes a reverse transcriptase domain [15]. It probably cleaves the target DNA to initiate reverse transcription and integration by a mechanism used by other non-LTR retrotransposons and group II introns [16].

In addition to the variety of opportunistic mobile genetic elements, the GIY-YIG domain has been found in enzymes involved in housekeeping processes. It is present in the bacterial UvrC family of nucleases, which participate in the nucleotide excision repair (NER) pathway by incising the damaged DNA strand on both sides of the damage [17]. The N-terminally located GIY-YIG domain is involved in cleavage on the 3' side, while an unrelated, C-terminally located domain is involved in the cleavage on the 5' side [18]. Recently, a crystal structure of the N-terminal domain of UvrC has been solved [19], revealing essentially the same three-dimensional fold as in the case of I-TevI [6], but with an additional helix at the C-terminus and different packing of helices against the central β-sheet. The GIY-YIG domain has been also identified in Cho, another endonuclease involved in NER in *E. coli*, which is capable of making the 3' incisions only, but is able to efficiently incise damaged DNA presenting bulky lesions that are poor targets for the N-terminal domain of UvrC, and allow the C-terminal domain of UvrC to complete the repair by making the 5' cut [20]. A GIY-YIG domain related to that in Cho has been identified in Mycobacteria, where it is fused to the homolog of ε 3' exonuclease, a proof-reading subunit of the DNA polymerase III holoenzyme. It was proposed that the exonuclease domain would digest the damaged DNA strand from the incision made by the GIY-YIG domain in the 3' direction through the lesion, to leave a 2' OH end as a primer for repair synthesis [21]. The GIY-YIG nuclease domain is also present in the Slx1 protein, a subunit of a eukaryotic yeast heterodimeric structure-specific endonuclease that is involved in the maintenance of rDNA copy number by stimulation of recombination of arrested replication forks via single-strand cleavage on the 3' side of the junctions [22,23]. A family of prokaryotic homologs of Slx1 have been also identified and predicted to be involved in DNA repair [24].

As outlined above, the GIY-YIG domain has been implicated in a variety of cellular processes involving DNA cleavage, from self-propagation with or without introns, to restriction of foreign DNA, to DNA repair and maintenance of genome stability. However, to date the phylogenetic history of GIY-YIG nucleases has not been studied and it is not clear how and when they got involved in these processes and how they should be classified in terms other than phenotypic (i.e. function). Besides, a detailed sequence and structural analysis of the GIY-YIG superfamily has not been made and it is not known if the current catalogue of members is complete or if there are many more potential members among the uncharacterized proteins in the databases. Therefore, we decided to carry out a systematic search for new potential members of the GIY-YIG superfamily, followed by comparative analysis aiming at the first comprehensive evolutionary classification of these important enzymes.

## Results and discussion

### Identification of new candidate GIY-YIG nuclease families with profile HHMs searches

In order to carry out a systematic search for new GIY-YIG nucleases, we prepared a set of multiple sequence alignments corresponding to previously identified families: UvrC, Slx-1, I-TevI, Penelope element, and REases. For each family we generated a profile HMM that included the sequences and predicted secondary structure (see Methods). These profile HMMs were compared with HHsearch [25] to a database of profile HMMs corresponding to multiple sequence alignments obtained from the COG, KOG, and PFAM databases, also with predicted secondary structures (see Methods for details). Based on the results of the HMM-HMM comparison, and in particular the list of "hits" with significant P-value (< 0.0001) and scores (>15.0), we generated a preliminary list of candidate GIY-YIG subfamilies. The preliminary candidates were validated by reciprocal HHsearches against the database comprising both the initial query profile HMMs as well as all the other COG, KOG, and PFAM profile HMMs. If a region of sequence that initially seemed to be similar to GIY-YIG enzymes displayed significant similarity to some other family, unrelated to GIY-YIG enzymes, then a given family was regarded as a false positive and was not further analyzed. Each candidate family, for which the relationship to known GIY-YIG sequences was confirmed by reciprocal searches, was also analyzed by fold-recognition (FR) methods to evaluate its compatibility with the known GIY-YIG structures (or detect cases, where some other, unrelated structure appeared to be a better template). Finally, comparative models were built for the parts of the sequence aligned by the FR methods to the template structures and the sequence conservation was analyzed in the structural context to detect potential active site residues (see below). Ultimately, our analysis revealed that COG1833 annotated as "uncharacterized ancient conserved region, predicted EndoIII-related endonuclease" and COG3680 annotated as "uncharacterized bacterial conserved region" are related to the GIY-YIG nucleases. The presence of the GIY-YIG domain in members of COG1833 had been already described [17]. However, to our best knowledge, its identification in COG3680 has not yet been reported in the literature.

### Alignment and preliminary classification of the GIY-YIG superfamily

In order to identify further new members of the GIY-YIG superfamily, which might have not been included in the COG or PFAM databases, we have carried out PSI-BLAST searches starting with representative sequences of all families (including COG1833 and COG3680). Searches were initially run with a stringent e-value threshold of 1e^-35 ^until convergence (to obtain confident alignments and more robust profiles) and later the threshold was relaxed to the value corresponding to 0.01 times the score of the first false positive and searches were continued until convergence. All sequences retrieved from all the PSI-BLAST runs were pooled together. After removing the duplicated entries, different isoforms of the same gene and a few obvious false positives, the final database of known and putative GIY-YIG members comprised 765 sequences from all major phylogenetic taxons: Bacteria, Archaea, Eukaryota, as well as viruses and Eukaryotic organelles. All these sequences were aligned using MUSCLE [26] to confirm the presence of a bona fide GIY-YIG motif and other characteristic features of this superfamily, and to define the boundaries of the nuclease domain. In the case of families known to include additional domains or long extensions of unknown structure, these regions were removed prior to the alignment. The initial alignment of the GIY-YIG domains was obtained automatically and subsequently refined by hand, based on the superposition of structures available for representatives of different families, analysis of alignments between different profile-HMMs and alignments proposed by FR methods during the first stage of this analysis (see above). For problematic sequences that would not align well with any particular family we carried out additional FR analyses and built comparative models (see Methods).

**Figure 1:**
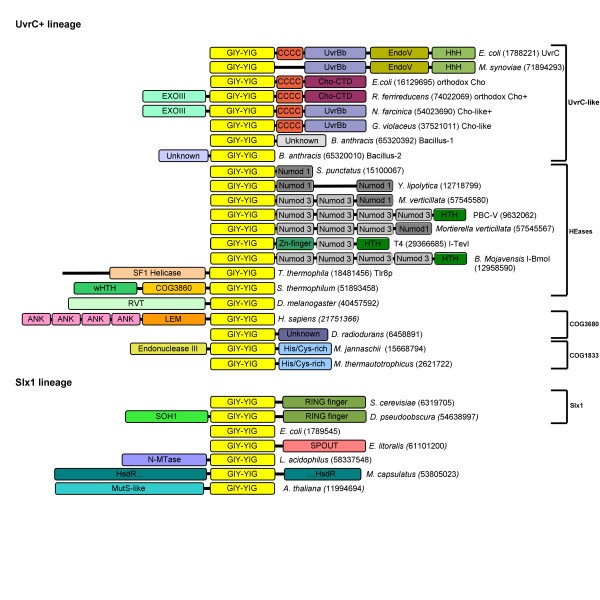
**Domain architectures observed in the GIY-YIG superfamily. **Numbers in round brackets indicate NCBI gene identification (GI) numbers of representative members of proteins sharing domain architecture. All representatives are divided into presumably monophyletic groups according to the sequence clustering. Light yellow blocks indicate the common GIY-YIG domain. Other domain abbreviations are: ANK=ANKRD41, ankyrin repeat domain 41; LEM, nuclear membrane-associated proteins domain; His/Cys-rich, histidine- and cysteine-rich conserved region; RVT, reverse transcriptase; CCCC, region with four conserved Cys residues; UvrBb, UvrB-binding domain; EndoV, Endonuclease V-like nuclease domain; Cho-CTD, C-terminal domain found in Cho and Cho-related proteins; EXOIII, exonuclease domain in the α and ε subunits of DNA-polymerase; UNKNOWN, different conserved domains of unknown function; SOH1, component of the RNA polymerase II transcription complex in *S. cerevisiae*; N-MTase, predicted DNA or RNA or protein MTase acting on exocyclic amino groups in bases or amino acids.; HsdR, restriction subunit of a putative Type I RM system (the GIY-YIG domain is inserted at position ~800); Numod1-3, conserved DNA-binding domains of homing endonucleases; HTH, Helix-turn-helix; wHTH, winged-helix-turn-helix; COG3860. SF1 Helicase, putative Superfamily 1 helicase domain.

In the process of delineation of precise boundaries of the GIY-YIG domain we have also carried out fold-recognition analysis for the sequences outside the nuclease domain. Figure 1 shows the representative architectures of GIY-YIG superfamily members, revealing that in majority of cases, the nuclease domain is associated with other domains fused either N- or C-terminally. The most interesting cases of domain fusions are described in detail in the following sections of the article.

**Figure 2:**
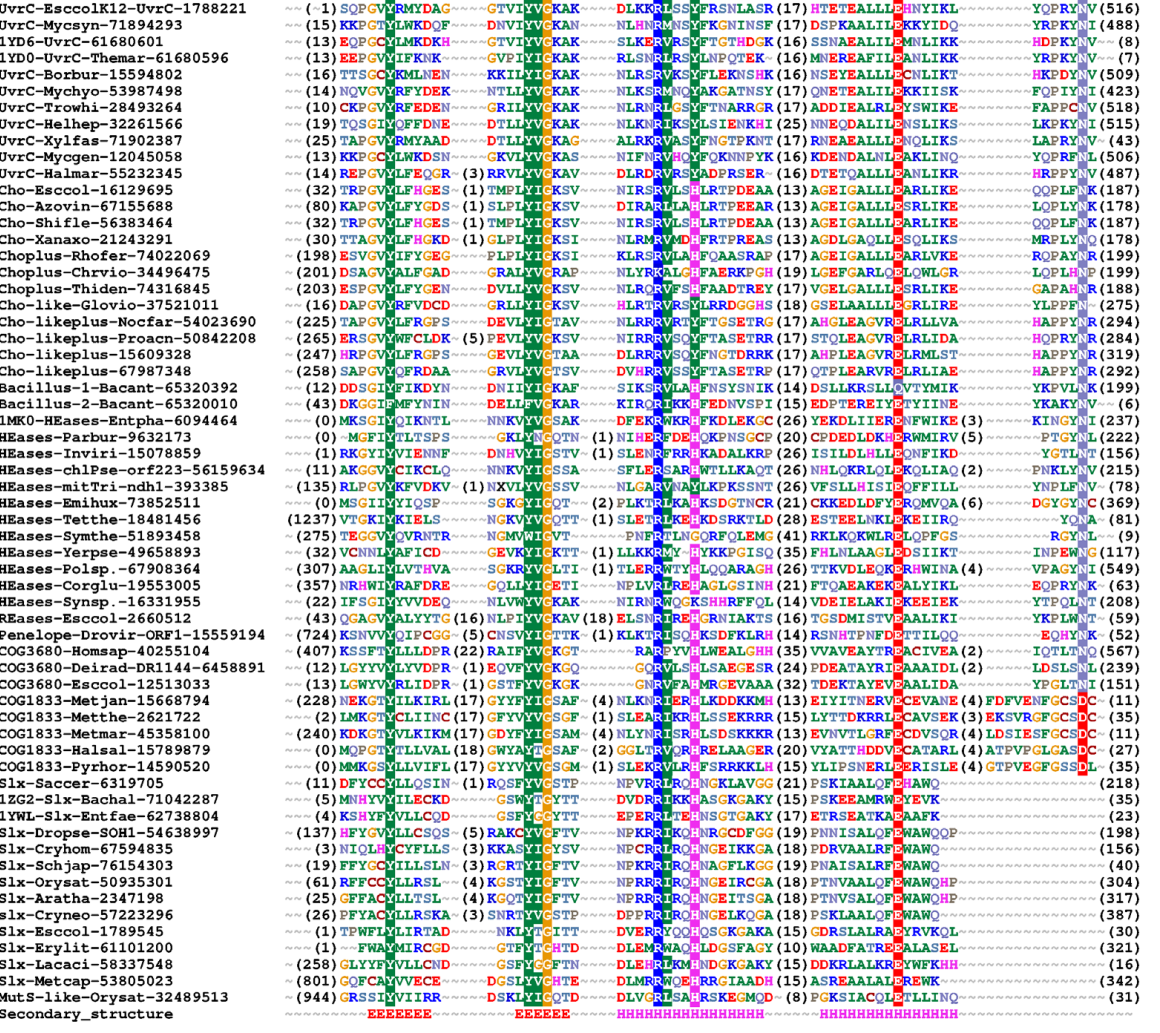
**Multiple sequence alignment of 61 selected representatives of the GIY-YIG superfamily. **Sequences were selected from each family (UvrC, Cho, Cho+Exo, Cho-like, Cho-like+Exo, Bacillus-1, Bacillus-2, HEases, REases, Penelope, COG3680, COG1833, Slx, MutS-like) to cover diversity of known structures and functions. Sequences are denoted by the species' name, the NCBI gene identification (GI) number and the PDB code (if applicable). Additionally sequences are grouped by families listed above. The variable termini and insertions are not shown; the number of omitted residues is indicated in parentheses. Amino acids are colored according to the physico-chemical properties of their side-chains (negatively charged: red, positively charged: blue, polar: magenta, hydrophobic: green). Conserved residues are highlighted. Secondary structure elements determined for the archaetypal member of the superfamily, I-TevI, are shown as H (helices) and E (strands).

Figure 2 shows the refined alignment of the GIY-YIG domain. The earlier analysis carried out for I-TevI and its closest homologs [1] identified the presence of five conserved motifs (A-E). Our analysis reveals that on the level of the whole GIY-YIG superfamily the bipartite motif A "GIY-YIG" should be split into two boxes, that motif "C" is not conserved between different families, and that motif "E" can be completely absent (as in the Slx-1 family) or have the conserved Asn residue (previously thought to be invariant) to be substituted by Asp (as on COG1833).

Based on the multiple sequence alignment we attempted to infer the phylogenetic tree of the GIY-YIG superfamily using the Neighbor Joining and Maximum Likelihood methods and several alternative models of evolution, with and without correction for mutational saturation (see Methods). Similar analyzes were carried out for the dataset reduced to 100 representatives from all major families (data not shown). Unfortunately, none of the trees we obtained could be considered as reliable according to the Shimodaira-Hasegawa test or the bootstrap criterion (data not shown). Although most families were found to produce monophyletic branches, COG3680 exhibited a tendency to split into two or more parts that grouped together with different families. Besides, the mutual position of different branches was found to vary significantly between trees calculated with slightly different parameters. Likewise, no stable trees could be obtained from the subsection of the alignment limited to the conserved motifs, e.g. absolutely reliable regions, perhaps due to the insufficient number of compared positions (data not shown).

Because no unique tree could be inferred based on the information contained in the multiple sequence alignment of the GIY-YIG domains, we decided to carry out a more "fuzzy" classification based on the clustering of pairwise sequence similarities, using CLANS [27]. We have experimentally found that the P-value thresholds of 10^-3 ^(for the full-length sequences) and 10^-2 ^(for the GIY-YIG domains) produced qualitatively best results. More stringent values caused disconnection of the most diverged sequences, while more permissive values caused over-compaction of the whole dataset into a single cluster with only a few outliers.

**Figure 3:**
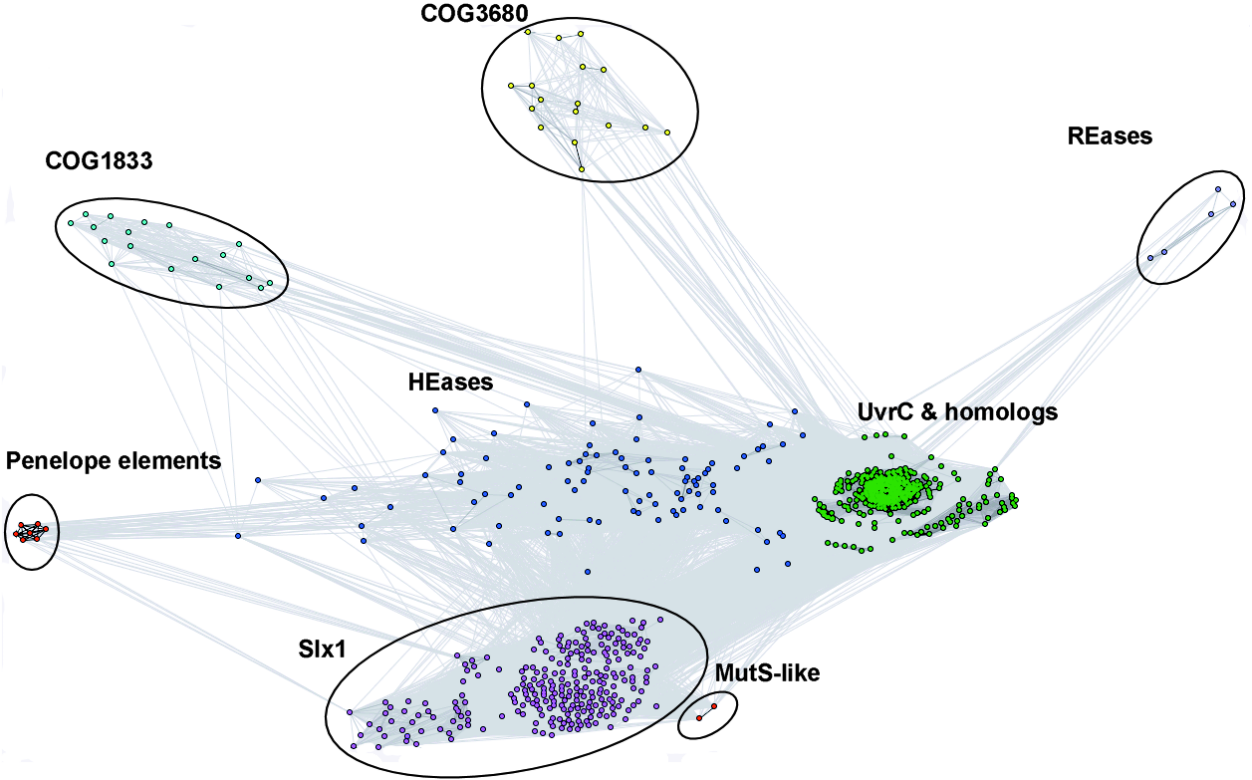
Two-dimensional projection of the CLANS clustering results obtained for the full-length GIY-YIG sequences.

**Figure 4:**
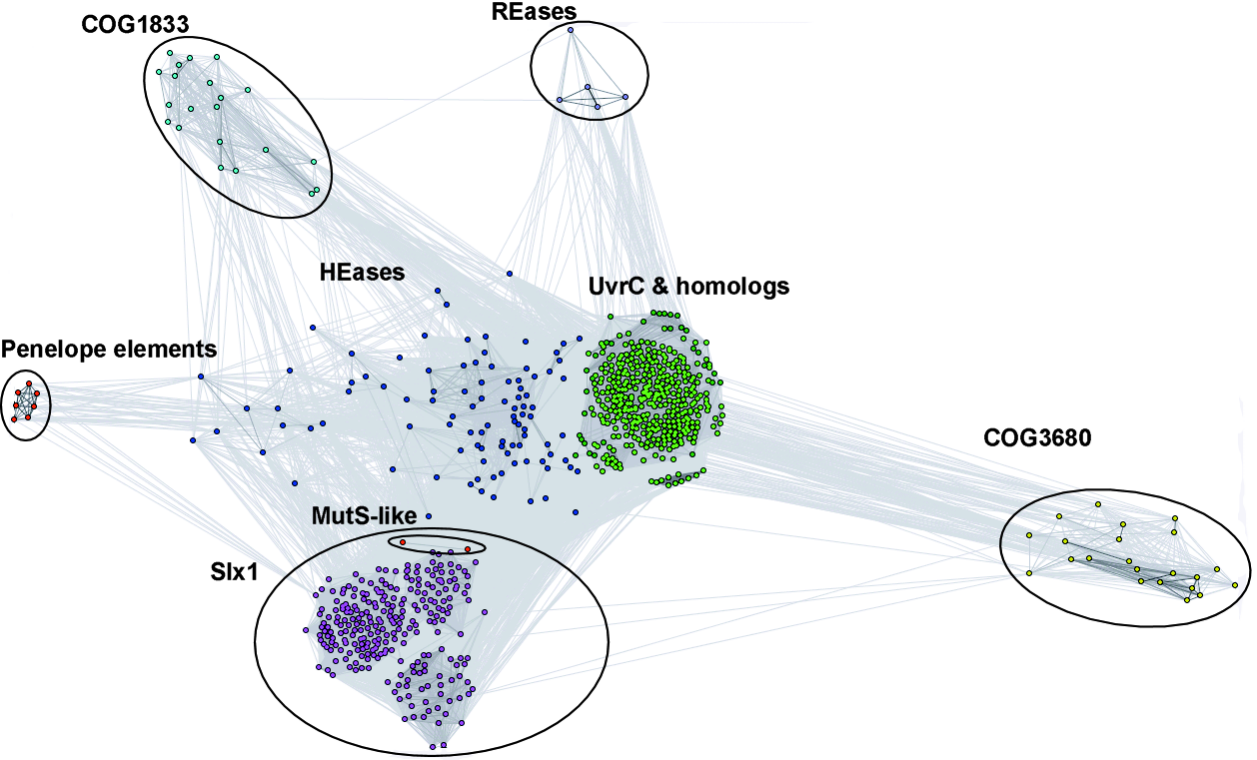
Two-dimensional projection of the CLANS clustering results obtained for the GIY-YIG domains isolated from sequences clustered in Figure 3.

Figures 3 and 4 show a representative 2D projection of "sequence clans" of the full-length sequences and the isolated GIY-YIG domains, respectively, obtained after several independent minimizations, starting with random distribution of sequences. The clustering was also carried out for the "purged" dataset, in which the number of representatives of each family was reduced to at most 20 selected most diverged members (data not shown). All these analyzes revealed a relatively similar, but complex pattern of relationships in the GIY-YIG superfamily, with very close as well as very distant similarities within and between different families, which can explain the failure of traditional phylogenetic methods.

The clustering reproduced all the groupings corresponding to the originally defined COGs, but also revealed additional interesting relationships. In particular, all analyzes reproduced a "supercluster" comprising the UvrC/Cho group (Bacteria and some Archaea) and HEases related to I-TevI and their relatives (viruses, Bacteria, and organelles), surrounded by well-resolved and weakly interconnected clusters corresponding to the families of Slx-1 (Eukaryota, and their orthologs from Archaea and Bacteria, as well as viruses), REases (Proteobacteria and *Deinococcus*), COG1833 (Archaea and Proteobacteria), and COG3680 (Bacteria and Eukaryota). Interestingly, proteins from the Penelope elements (Eukaryota), which by themselves form a small dense cluster, are connected to the HEase cluster by a dispersed "cloud" of strongly diverged sequences.

Based on the results of clustering (Figures 3 and 4) and detection of characteristic domains (Figure 1), we classified all members of the GIY-YIG superfamily into families and subfamilies (Table 1).

**Table 1:** 

**Family**	**Subfamily**	**No. of members**	**A**	**B**	**E**	**V**	**O**	**Characteristic structural features**
UvrC-like	UvrC	318	8	310	-	-	-	CTD: C4-UvrBb-EndoV-HhH
	Cho	20	-	20	-	-	-	CTD: C4-ChoCTD
	Cho+Exo	18	-	18	-	-	-	CTD: C4-ChoCTD, NTD: ExoIII
	Cho-like	1	-	1	-	-	-	CTD: C4-UvrBb
	Cho-like+Exo	11	-	11	-	-	-	CTD: C4-UvrBb, NTD: ExoIII
	Bacillus1&2	10	-	10	-	-	-	NTD&CTD: unknown conserved domains
HEases	orthodox	91	-	10	-	27	54	CTD: Different patterns of NUMODs
	unorthodox	12	-	9	3	-	-	no NUMODs, other domains sometimes present
COG1833	orthodox	20	15	5	-	-	-	CTD: His/Cys-rich
	EndoIII-fusion	2	2	-	-	-	-	CTD: His/Cys-rich, NTD: Endonuclease III
COG3680	Eukaryota	15	-	-	15	-	-	NTD: ankyrin repeats, LEM
	Bacteria	9	-	9	-	-	-	CTD: unknown conserved domain
Penelope		10	-	-	10	-	-	NTD: RVT
REases		5	-	5	-	-	-	GIY-YIG with insertions and terminal extensions
Slx1	orthodox	30	-	-	30	-	-	no C-terminal Asn, CTD: RING finger
	MutS-fusion	2	-	-	2	-	-	no C-terminal Asn, NTD: MutS domain
	non-Eukaryota	208	2	191	-	15	-	no C-terminal Asn
Total:		782	27	599	60	42	54	

### Evolutionary relationships between and within GIY-YIG families

#### HEase/UvrC supercluster

**Figure 5:**
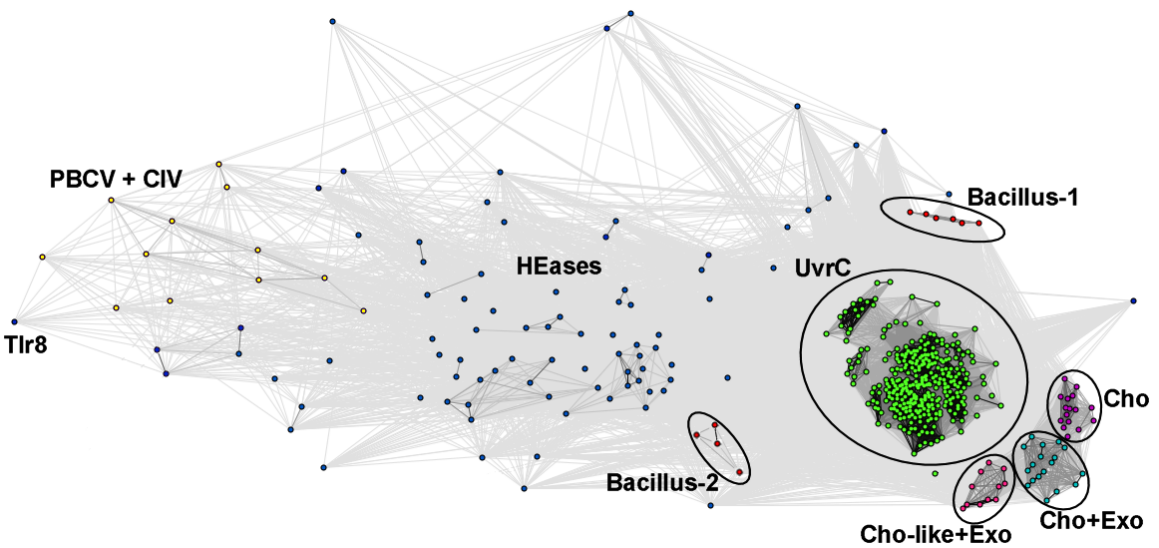
**Two-dimensional projection of the CLANS clustering results obtained for the full-length sequences of the "supercluster" **Sequences were taken from central "supercluster" in Figures 3 and 4. Proposed subfamilies are colored and labeled: HEases – blue, orthodox UvrC (with EndoV domain) – green, orthodox Cho – magenta Cho-like+Exo domain – light pink, Cho+Exo domain – cyan, Bacillus-1 and Bacillus-2 – red. Additional labels: PBCV-1 virus and Chilo iridescent virus – yellow, Tlr8 from *Tetrahymena thermophila *– black

To elucidate the relationships within the central "supercluster" comprising HEases, UvrC, and related sequences, we carried out a separate CLANS analysis using the 10^-3 ^P-value threshold. The results show that the central cluster of orthodox UvrC proteins is surrounded by satellite clusters of UvrC-like protein lacking the EndoV domain, Cho homologs, and a dispersed "cloud" of HEases and their relatives (Fig 5).

Analysis of the domain distribution among UvrC and Cho homologs (Figure 1) reveals a complex pattern of presence or absence of additional domains, which exhibit interesting phylogenetic correlations. The "orthodox" UvrC, such as the well-studied representative from *E. coli *are characterized by different C-terminal domains fused C-terminally to the GIY-YIG domain via a common Cys-rich domain: UvrC has a UvrB-binding domain (UvrBb), EndoV-like nuclease domain, and a HhH domain [28]. UvrC is present in nearly all bacterial species, with the exception of obligate pathogens and endosymbionts with reduced genomes, *Ehrlichia, Wigglesworthia *and *Buchnera *[29,30]. In Archaea UvrC is completely absent from Crenarchaeota and present only in some Euryarchaeota, namely Halobacteriales and Methanosarcinales, as well as in *Methanothermobacter thermautotrophicus *and *Methanococcus maripaludis*, although it is absent from its close relative *Methanocaldococcus jannaschii*. In the "orthodox" Cho represented by the protein from the *E. coli*, the UvrBb-EndoV-HhH module is replaced with a unique domain that shows no similarity to other protein families (data not shown). This variant is present only in Gammaproteobacteria, but its distribution is patchy, e.g. it is often absent from close relatives of species that possess it. It has been reported that some Cho homologs (e.g. in *Mycobacterium*) have an additional ExoIII domain fused N-terminally to the GIY-YIG domain [21]. We found that these proteins can be further divided into the variety present only in a few Betaproteobacteria that possesses the Cho-specific C-terminal domain, and the variety specific to Actinobacteria, which instead possesses the UvrC-like UvrBb domain. We also found a variant that possesses the UvrBb domain and lacks the ExoIII domain (i.e. similar to the orthodox UvrC devoid of the EndoV and HhH domain) in *G. violaceus *(GI: 37521011). In pathogenic species of *Bacillus *we found two different subfamilies of remote homologs of UvrC, one termed Bacillus-I, in which the whole C-terminal region (including the Cys-rich domain) is replaced by another unknown domain (e.g. *Bacillus anthracis*, GI: 65320392), and the other termed here Bacillus-II, which has only an N-terminal extension (e.g. *B. anthracis*, GI: 65320010). In several species of *Mycoplasma *(e.g. *Mycoplasma synoviae*, GI: 71894293) we also found UvrC variants with a deleted Cys-rich domain but all other domains retained. Our findings contradicts the proposal of Van Houten and co-workers that mycoplasmas and *Borrellia burgdorferi *lack UvrC and possess only Cho [21] and suggest that these species lack Cho, while the disputed members of the GIY-YIG superfamily (e.g. GI: 15594802 in *B. burgdorferi*) are orthologs of UvrC.

In order to reconcile the evolutionary history of UvrC/Cho proteins, we resorted to two complementary approaches. First, we calculated a maximum likelihood tree for the alignment of GIY-YIG domains. Second, we calculated a maximum parsimony tree for the full-length sequences, following the conversion of different types of domains into equivalent characters. Both methods provided slightly different solutions depending on the parameters used (data not shown). The common features, however, allowed us to infer that UvrC was the ancestral form, which underwent duplication, giving rise to the truncated Cho-like form (without EndoV-HhH), which in some bacteria acquired ExoIII domain. This form has then replaced the UvrBb domain with another domain, giving rise to the "orthodox" Cho, which is some cases (e.g. in *E. coli*) has lost the ExoIII domain. The forms present in pathogenic Bacillales evolved from a duplicate of UvrC (possibly, but not necessarily from Cho) that either replaced the C-terminal segment with a completely different domain or lost the C-terminal segement and acquired an N-terminal extension.

The dispersed "cloud" of HEases and their relatives contains a number of sequences, typically containing a variety of NUMOD domains [31] fused C-terminally to the GIY-YIG domain. In addition to the archetypal members from phages (e.g. I-TevI), the HEase cluster includes also bacterial sequences. Genomic neighborhood analysis revealed that most of them are located within known mobile genetic elements like transposones or prophages or close to proteins characteristic for such elements (e.g. transposase, terminase, head portal protein etc.). Thus, the current bacterial hosts have most likely acquired members of the HEase cluster from phages. Interestingly, some of these proteins contain additional regions/domains, not found in the orthodox NUMOD-containing members. For instance a protein from Actinobacterium *Symbiobacterium thermophilum *(GI: 51893458) contains the N-terminally fused LuxR module, comprising the wHTH domain and an uncharacterized domain annotated as COG3860. Some relatives of I-TevI contain other, unrelated domains, for which we could not reliably detect any homologs or to predict the three-dimensional folds. (e.g. hypothetical proteins Ncgl1730 from *Corynebacterium glutamicum*, GI: 19553005, sll0664 from *Synechocystis sp*., GI: 16331955, and api45 from *Yersinia pseudotuberculosis*, GI: 49658893)

Given the close relationship between the UvrC/Cho family and the HEases, it is tempting to speculate that the latter evolved from one of the duplicated copies of UvrC that acquired such sequence-specific DNA-binding domain (e.g. among the NUMOD families [31]) that targeted the GIY-YIG domain to cleave the alleles that lacked the nuclease-encoding gene. Among the fully sequenced genomes, the presence of HEase coincides with UvrC paralogs (i.e. non-orthodox copies in addition to the orthodox UvrC) only in the aforementioned pathogenic Bacillales. However, the currently available data on phylogenetic distribution and mutual similarity of genuine HEases characterized by NUMOD domains and the non-orthodox paralogs of UvrC family are too sparse to delineate the putative functional transformation between the "house-keeping" UvrC-like and "selfish" HEase-like life styles in the GIY-YIG superfamily. Interestingly, we found no HEases from the GIY-YIG superfamily in Alphaproteobacteria. This suggests that Eukaryotic mitochondria could have acquired these nucleases by some other route than via the vertical descent from the free-living Alphaproteobacterial ancestor. For instance, Eukaryotic mitochondria, as well as chloroplasts (e.g. in *Pseudendoclonium akinetum *[32]) could have been invaded by GIY-YIG HEases that parasitized the pathogenic bacteria living in intimate contact with their host.

Among new members of the GIY-YIG superfamily associated with the UvrC/HEase supercluster, particularly interesting are the most diverged sequences that appear to connect HEases with Penelope elements: Tlr8Rp (GI: 18481456) – a 1405 aa long protein encoded by a recently characterized Tlr element found in a ciliated Protozoan *Tetrahymena thermophila *[33] and a few sequences from the Phycodnavirus PBCV-1 DNA virus (GIs: 9632062, 9632173, 9631883, and 9631703) and the Iridovirus Chilo iridescent virus (CIV) (GIs: 2738435 and 15042176). The FR analysis of the viral proteins confirmed the presence of the N-terminally located I-TevI-like GIY-YIG domain (1ln0, e.g. INBGU score 67.6, PCONS score 2.279), and detected the presence of C-terminal NUMOD domains that were also reported in I-TevI and other HEases [31]. On the other hand, Tlr8 shares with these proteins only the I-TevI-like GIY-YIG domain in the C-terminus (aa 1240–1340) (PCONS2, score 1.42). Tlr8p contains also a putative Superfamily 1 helicase domain (aa ~800–1200). Its N-terminal region (aa 1–140) exhibits relatively high propensity to form the coiled-coil structure. The central region (aa 140–800) exhibits a pattern of secondary structures typical for well-folded globular domains (data not shown), however we could not detect its relationship to any previously characterized protein families or structures. Tlr elements belong to a family of approximately 30 micronuclear DNA sequences that are efficiently eliminated from the developing somatic macronucleus, when chromosomal breakage occurs at hundreds of specific sites (chromosomal breakage sequences, CBS). Interestingly, some Tlr elements were already found to possess insertion sequences encoding putative HEases, comprising a HNH nuclease domain in the N-terminus and an APETELA2 DNA-binding domain in the C-terminus [34]. It will be interesting to determine if Tlr8p is active as a nuclease and whether it may target CBS or a related sequence.

The presence of common NUMOD domains suggests that these PBCV-1 and CIV acquired the I-TevI-like HEase from phages by horizontal gene transfer. These acquisitions could have been independent, but since Phycodnaviruses and Iridoviruses are believed to be evolutionarily related [35], transfer to the common ancestor of Phycodnaviruses and Iridoviruses followed by vertical descent cannot be excluded. Alternatively, these viruses could have inherited the HEase from the common ancestor of phages and dsDNA viruses, although this would require massive losses of the HEase from other phage and viral lineages. Interestingly, PBCV-1 infects Eukaryotic unicellular Chlorella-like algae that live endosymbiotically within the ciliate *P. bursaria *[36], while CIV is pathogenic for a variety of insect larvae. This suggests that the Penelope elements could have acquired the GIY-YIG domain within the insect cell infected by the Iridovirus, while the Tlr8p element could have acquired its GIY-YIG domain within the nucleus of the ciliate cell infected by the Phycodnavirus. In both cases the GIY-YIG domain has apparently lost the associated HEase-like NUMOD domains.

#### COG3680

COG3680 groups together functionally uncharacterized and not annotated proteins from bacteria, which to our knowledge have not been reported as members of the GIY-YIG superfamily in the literature. Our profile-HMM analysis identified this family as a relative of the GIY-YIG domain from the UvrC family (P-value 10^-5^). This prediction was also confirmed by the FR analysis, which identified the GIY-YIG domain as the best template for COG3680 sequences: I-TevI (1ln0): INBGU score 47.26, UvrC (1yd0/1ycz) SAM-T02 score 0.0038, PCONS2 consensus score 1.513. PSI-BLAST searches revealed that five members of COG3680 have homologs in several other bacteria as well as in Eukaryota, including the ANKRD41 (ankyrin repeat domain 41) protein from *Homo sapiens *(GI: 40255104). ANKRD41 bears the Gene Ontology annotation "kinase activity", but closer inspection of the original database entry (GI: 21751365) reveals that this functional annotation was based on the finding that this sequence is "weakly similar to cyclin-dependent kinase 4 inhibitor A". The Eukaryotic homologs are much longer than their Prokaryotic relatives, due to an N-terminal extension of 20–120 aa, which includes the region of approximately 3–4 (meta: 12261) ankyrin repeats (according to the secondary structure prediction for ANKRD41) that apparently have lead to the misleading "kinase" misannotation. Using FR analysis we have also found that the ankyrin repeats and the GIY-YIG domain in Eukaryotic members of the COG3680 family are separated by the LEM domain (aa 370–410 in ANKRD41). Ankyrin repeats mediate protein-protein interactions in very diverse proteins, including protein kinases and transcription factors [37]. The LEM domain was identified in nuclear membrane-associated proteins, including lamino-associated polypeptide 2, emerin, and MAN1 [38]. It was shown that LEM domains can be involved in protein- or DNA-binding [39]. Such composition of domains suggests that Eukaryotic members of COG3680 are involved in interactions with multiple partners, which implies an important cellular function. Analysis of the phylogenetic distribution reveals that members of COG3680 are present only in Metazoa, and a few pathogenic bacteria such as Neisseriaceae, pathogenic strains of Enterobacteriaceae (e.g. *E. coli *O157:H7 EDL933) and *Haemophilus *(e.g. *H. influenzae *R2866 or 86-028NP) but not in their non-pathogenic relatives. The only exception to this rule is the presence of the COG3680 member in non-pathogenic *Deinococcus radiodurans *R1. Based on the presence of the newly detected GIY-YIG domain, we predict that all members of COG3680 are nucleases, possibly engaged in DNA repair or recombination. It will be interesting to study the link of bacterial members with pathogenicity.

#### COG1833

COG1833 includes a few functionally uncharacterized proteins (~150 aa) mainly from Archaea (both Euryarchaeota and Crenarchaeota). Members of COG1833 are absent only from Thermoplasmatales, which in general possess no detectable members of GIY-YIG superfamily at all. A few members of COG1833 present in Bacteria such as *Thermotoga maritima *or *Methylococcus capsulatus*, could have been acquired by horizontal gene transfer from extremophilic Archaea dwelling in the same environment. The GIY-YIG domain was originally identified in some of these proteins by Aravind et al. [17], who also noted the presence of a "UvrC-endonuclease III fusion" in MJ0613 from *Methanocaldococcus jannaschii *(GI: 15668794). Curiously, the prediction of the GIY-YIG domain in proteins now classified as COG1833 is not at all reflected in their current annotations in databases. Rather, many members of COG1833 are annotated as "putative endonuclease III", even though they lack the endonuclease III domain. Our analysis revealed that endonuclease III is N-terminally fused to the GIY-YIG domain only in MJ0613 and its homolog from a closely related species *Methanococcus maripaludis *(GI: 45358100, MMP0538). In other species that contain members of COG1833, endonuclease III is encoded by a separate gene. Thus, we suggest that the current database annotation of COG1833 is spurious and should be corrected, although the fusion of two nuclease domains in Methanococcales does suggest some functional cooperation. Endonuclease III is a bifunctional enzyme N-glycosylase and apurinic/apyrimidinic(AP)-lyase, which excises damaged bases from the DNA and introduces a single-strand nick at the AP site from which the damaged base was removed. The fused variant of COG1833 or a complex between Endonuclease III and a "standalone" GIY-YIG domain of COG1833 could use the AP lyase and nuclease functions to cleave the DNA on both sides of the damage, in analogy to the action of a bifunctional nuclease UvrC. The GIY-YIG domain could also perform some other role, for instance to serve as an exonuclease that creates a single-stranded gap starting from the nick generated by the AP lyase of the Endonuclease III domain. It remains to be determined if COG1833 members are indeed involved in base excision repair (BER) or in any other DNA repair pathway. Interestingly, all members of COG1833 share a C-terminal extension (termed "meta-binding cluster" in ref. [17]) with semi-conserved Cys and His residues. We carried out FR and HHsearch analyzes separately for this region and could not find any significant similarities to known protein domains. The semi-conserved character of the potential metal-binding ligands suggests that they may be involved in stabilization of this additional (sub)domain rather than in catalytic activity.

#### Slx-1 cluster

Eukaryotic Slx-1 proteins are involved in the maintenance of the rDNA copy number [22]. In yeast, Slx1 acts together with an unrelated protein Slx4 [22,23], which has additional roles in the DNA damage response that are distinct from the function of the hewterodimeric Slx1-Slx4 nuclease [40]. The characteristic feature of the orthodox members of the Slx1 family is the presence of a C-terminal RING finger Zn-binding domain postulated to mediate protein-protein or protein-DNA interactions [41]. Interestngly, we found that the Slx1 ortholog from *Drosophila pseudoobscura *(GI: 54638997) contains an N-terminally fused SOH1 domain (HHSEARCH e-value 0). SOH1 is a component of the RNA polymerase II transcription complex in *Saccharomyces cerevisiae *and was reported to interact with factors involved in DNA repair and transcription, and hence it was proposed to serve to couple these two processes [42]. It will be interesting to determine if Slx1 from other species (e.g. in yeast) interact with the standalone SOH1, as this may reveal new links between maintenance of genome stability, DNA repair and transcription.

We found several new eukaryotic proteins that possess the GIY-YIG nuclease domain closely related to Slx1, but without the RING finger domain. A number of "hypothetical" proteins, such as are Chro.20460 from *Cryptosporidium hominis *(GI: 67594835), SJCHGC08377 from *Schistosoma japonicum *(GI: 76154303), OSJNBa0016A21.134 from *Oryza sativa *(GI: 50935301) or At2g30350 from *Arabidopsis thaliana *GI: 2347198 consist only of an Slx1-like GIY-YIG domain and intrinsically unstructured extensions of unknown function. It is possible that these unstructured segments are involved in interactions with other (so far unknown) proteins. In plants we have also found a small family of MutS homologs [43] with a C-terminally appended Slx1-like GIY-YIG domain, without the RING-finger, and with a predicted mitochondrial localization (GIs: 11994694 and 32489513). In *A. thaliana*, this protein is encoded by the *CHM *(chloroplast mutator) locus. It was found that Chm mutations lead to rearrangements in mitochondrial rather than chloroplast DNA and thereby affect mitochondrial gene expression and mitochondrial function, resulting in green and white variegation in leaves [44,45]. This suggests that the Chm protein could be involved in the maintenance of mitochondrial genome stability, in analogy to the orthodox eukaryotic members of the Slx1 family. The MutS family groups together several paralogous lineages of enzymes involved in DNA repair or recombination (review: [43]). One of these lineages (MutS2) contains proteins with a C-terminal nuclease domain from the Smr family, which has been shown to nick the DNA, albeit its role has not been elucidated in detail [46]. The structure of the Smr domain is unknown; it shows no particular similarity to the GIY-YIG domain. It will be interesting to determine if the Smr and GIY-YIG domains have a similar function in the context of their analogous fusions to members of the MutS family.

Prokaryotic orthologs of Slx1 have been identified by Aravind and Koonin and predicted to be involved in double-strand break repair [24]. Here, we report novel prokaryotic members of this family with new domain associations. In five species of Lactobacillales (GIs: 58337548, 42519373, 24379094, 23003819, and 48865048) we identified a GIY-YIG nuclease with an N-terminally appended Rossmann-fold methyltransferase (class-I MTase) domain [47,48], member of COG4123 annotated as "predicted O-methyltransferase". Our sequence analysis revealed a "NPPY" motif characteristic for N-MTases acting on nucleic acids or proteins [49], while we could detect no particular similarity with any O-MTases. We analyzed the genomic neighborhood of the Slx1-like prokaryotic nucleases and found further 5 examples where the ORF encoding a standalone GIY-YIG nuclease associated with a member of COG4123. This recurring association suggests that five fusion proteins arose from a conserved MTase-nuclease operon, similar to those observed in Type II restriction-modification (RM) systems. Thus, it is an attractive hypothesis (to be confirmed experimentally) that these newly discovered translational or transcriptional fusions are also involved in modification and cleavage of DNA. Another intriguing association of a Slx1-like domain with a RM system was found in the case of ORF MCA0838 from *Methylococcus capsulatus *(GI: 53805023). This ORF encodes a restriction (HsdR) subunit of a putative Type I RM system, comprising a typical tandem of AAA+ domains and a PD-(D/E)xK nuclease domain, with GIY-YIG domain inserted at position ~800. Strikingly, it has a very close homolog in *Deinococcus geothermalis *(GI: 66796591, 80% sequence identitiy), which completely lacks the GIY-YIG domain. It appears that the GIY-YIG domain has been very recently inserted into the HsdR subunit and may not be important for the nuclease function of this protein.

Interestingly, in *Erythrobacter litoralis *(GI: 61101200) we have also found a C-terminal fusion of a Slx1-like GIY-YIG domain with a MTase, but one with a completely different fold and function (SPOUT, class IV)[48,50]. So far SPOUT MTases have been only reported to act on RNA and never on DNA (review: [51]). In particular the domain found fused to the GIY-YIG nuclease appears to be an ortholog of the TrmH MTase, which modifies 2'-OH group of ribose in position 18 of tRNA [52]. This fusion in turn suggests that a prokaryotic ortholog of Slx1 may be involved in RNA metabolism.

### Evolutionary origin and phylogenetic history of GIY-YIG nucleases

**Figure 6:**
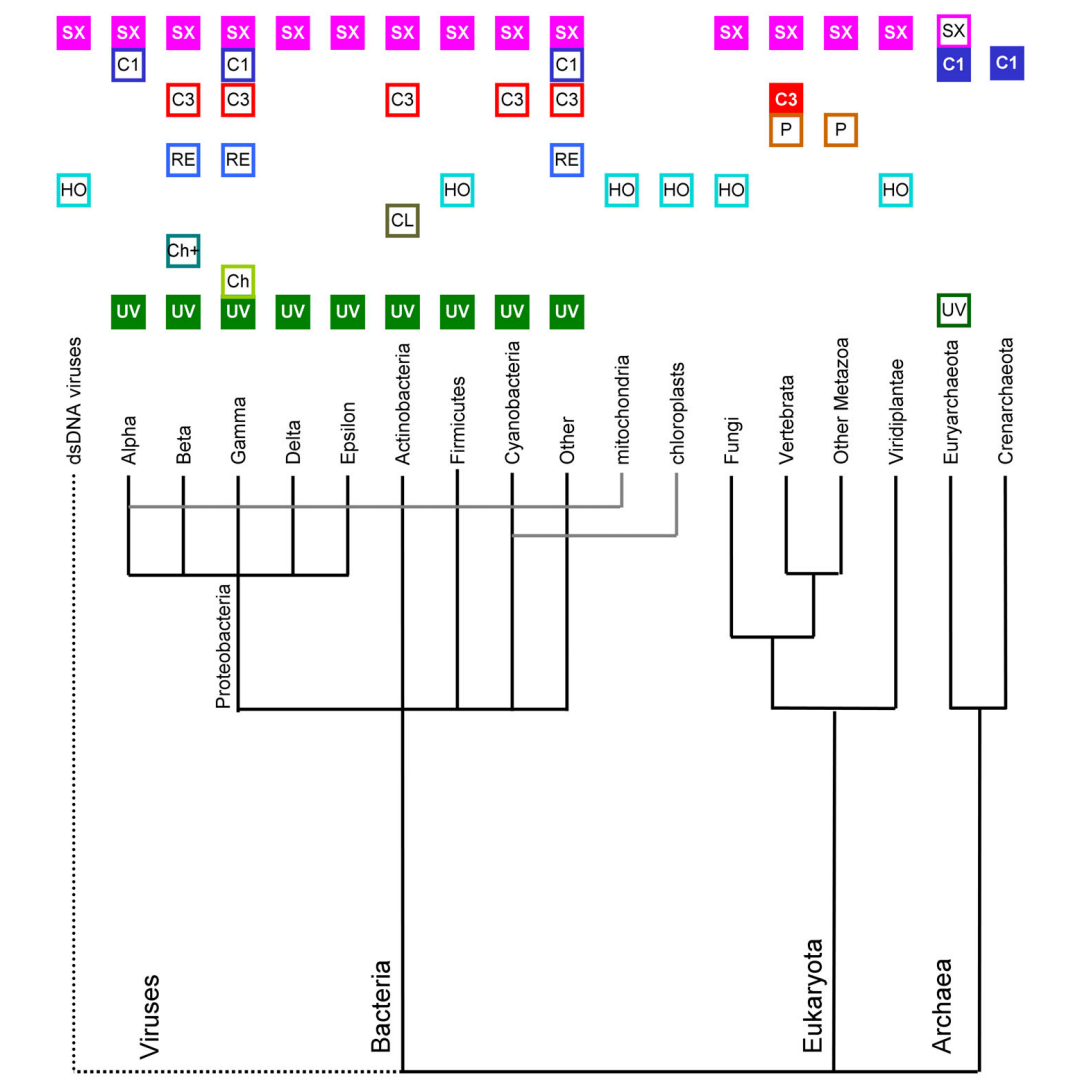
**Distribution of GIY-YIG nucleases from different subfamilies among the three Domains of Life. **Empty box indicates the presence of at least one family member in the corresponding taxon. Filled box indicates the presence of family members in >50% of fully sequenced genomes from the corresponding taxon. Abbreviations are: UV = UvrC, Ch = orthodox Cho, Ch+ = orthodox Cho+ExoIII, CL = Cho-like and Cho-like+ExoIII, HO = Heases, RE = REases, P = Penelope, C = COG3680, C1 = COG1833, SX = Slx1.

Based on the results of phylogenetic analyses for individual families as well as comparison of genomic distributions (Figure 6), we propose that the Last Universal Common Ancestor of all contemporary life forms (LUCA) contained at least two paralogous copies of the GIY-YIG superfamily nuclease, one related to Slx1 and the other to the ancestor of UvrC and possibly COG1833 and COG3680. The Slx1 family is represented in all three Domains of Life and the relationships between its members inferred from a phylogenetic tree (data not shown) suggest strong conservation and vertical descent from a common ancestral nuclease, with only a few obvious exceptions, where additional copies were generated by duplications and spread by horizontal gene transfers, often accompanied by fusions with additional domains involved in various aspects of nucleic acid metabolism (e.g. repair or methylation). On the other hand, the UvrC, COG1833, and COG3680 families are typical for Bacteria, Archaea, and Eukaryota, respectively. The distribution of UvrC members in Archaea, COG1833 members in Bacteria, and COG3680 members in Bacteria is irregular. They are restricted to only a few lineages, which often share a similar environment or life style (e.g. extremophily or pathogenesis), which strongly suggest acquisition by horizontal gene transfer. However, when the phylogenetic tree of the whole UvrC+ group is corrected for these obvious horizontal gene transfers, its topology exhibits good agreement with the species' tree (data not shown) arguing for a vertical descent from a common ancestor. Thus, we propose that the UvrC, COG1833, and COG3680 families are members of one orthologous lineage (hereafter termed the UvrC+ lineage) that acquired different auxiliary domains after (or during) the emergence of the three Domains of Life.

**Figure 7:**
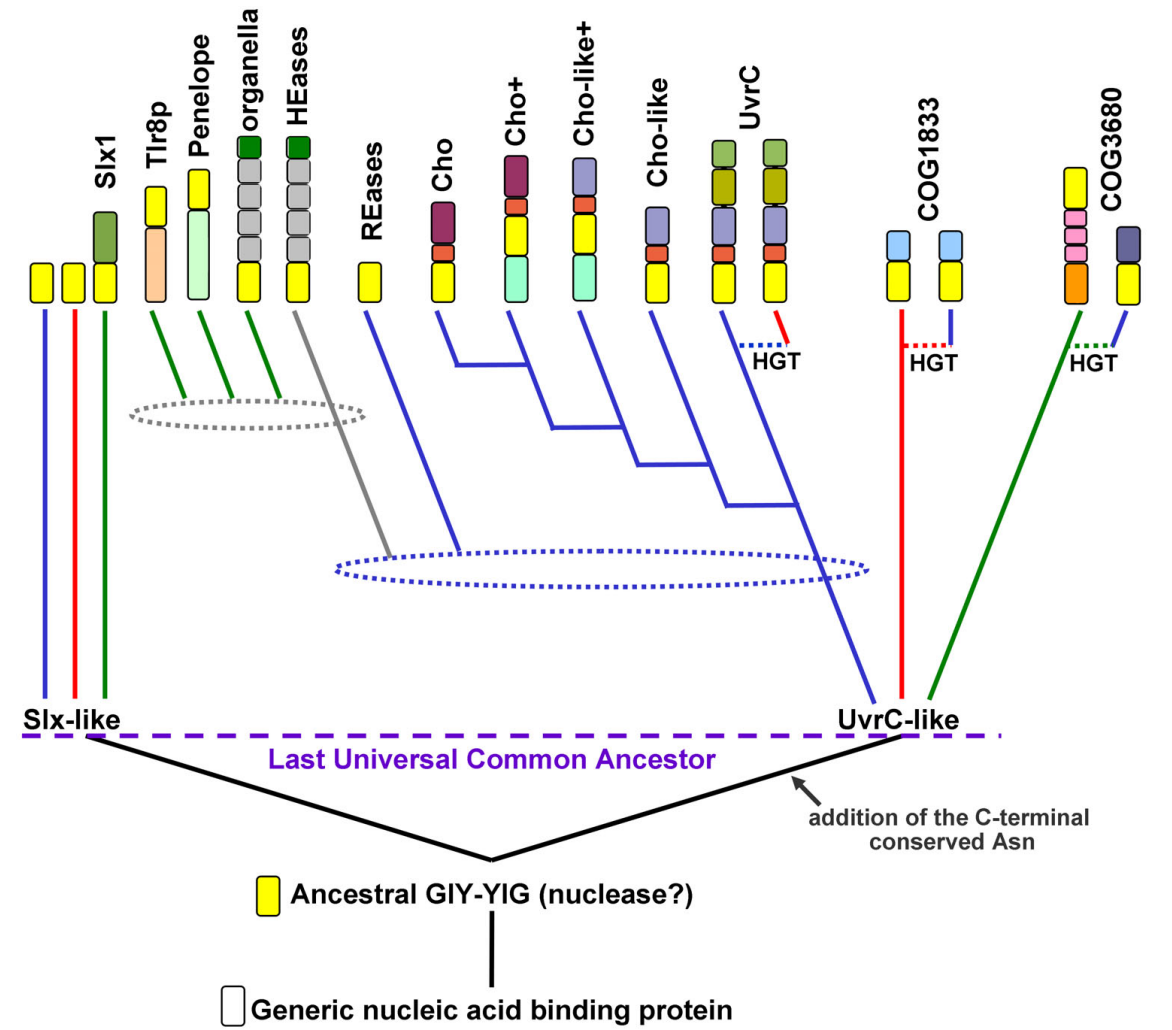
**The postulated phylogenetic tree of the GIY-YIG superfamily. **Only the major branches corresponding to subfamilies delineated in this work are shown. Colored blocks describe typicall domain architecture of corresponding family (the same as in Figure 1, however domain names are not shown). Blue, red, and green lines indicate bacterial, archaeal, and eukaryotic lineages. Dotted lines labeled 'HGT' indicate horizontal gene transfer events between different lineages. Dotted ellipses indicate the approximate time of intragenic duplications or other cases of horizontal gene transfer.

The hypothesis of ancient paralogy between the Slx1 and UvrC+ lineages is supported by sequence comparisons. First, the characteristic Asn residue in the C-terminal part of the GIY-YIG domain is completely absent in Slx1, but appears in nearly all members of the UvrC+ lineage, with the exception of COG1833, where it is replaced by a His-Cys-rich cluster. Second, the results of the CLANS analyses reveal evidently stronger clustering of COG1833 and COG3680 families with the UvrC family rather than with Slx1 (Figures 3 and 4). The orthologous groups corresponding to Slx1 and UvrC families were predicted to be present in LUCA by Ouzounis and coworkers [53]. On the other hand, neither Slx1 nor UvrC were found among the COGs predicted to be present in LUCA by Mirkin, Koonin and co-workers [54]. The discrepancy of these results is unclear to us, especially with respect to Slx1, whose common presence in all three domains very strongly suggests its presence in LUCA. With respect to UvrC, COG1833, and COG3680 families, neither of these analyses tested the possibility of an orthologous relationship between these lineages, hence their results are not directly comparable to ours. We favor the scenario, in which the ancestor of the postulated UvrC+ cluster was present in the LUCA as implied by the inference made by Ouzounis and coworkers [53], and gave rise to COG1833, and COG3680 by a vertical descent (Figure 7). In this scenario, the lack of COG1833 and COG3680 members from some Archaea and non-Metazoans can be explained by gene loss. However, we cannot exclude an alternative scenario, in which members of COG1833 and COG3680 were introduced to Archaea and Metazoa by horizontal transfer of a UvrC-like gene from Bacteria. In this scenario, the Slx1-UvrC+ duplication could have occurred either in LUCA or in the branch leading from the LUCA to the last common ancestor of Bacteria.

According to CLANS, the selfish members of the GIY-YIG superfamily from the REase and HEase clusters have also evolved from the UvrC+ lineage rather than from Slx1. However, their mutual relationship is unclear. REases from the GIY-YIG superfamily do not contain additional domains, but rather insertions and terminal extensions of the common core that increase the size of the nuclease domain and presumably build the scaffold for the DNA-binding site that recognizes short sequences with very high specificity [12,14]. However, they associate with DNA MTases, which protect the host genome from the cleavage of the target sites. On the other hand, the GIY-YIG domains in HEases are inherently non-specific and require fusions with multiple DNA-binding modules [31] to recognize their long targets. It is tantalizing to consider HEases as another type of RM systems, where "modification" that protects the DNA against the multiple rounds of cleavage is not the reversible methylation, as in the case of "classical" RM systems, but an irreversible insertion of the self DNA into the target site. It is possible that the small family of GIY-YIG REases evolved from HEases by reduction of the target site and association with the MTase of similar specificity. The possible close relationship of these two groups of GIY-YIG nucleases will be confirmed when the crystal structure of a GIY-YIG REase is determined, enabling quantitative assessment of the evolutionary distance by comparison of atomic coordinates, similarly to the study previously carried out for the nucleases from the PD-(D/E)xK superfamily [55,56].

**Figure 8:**
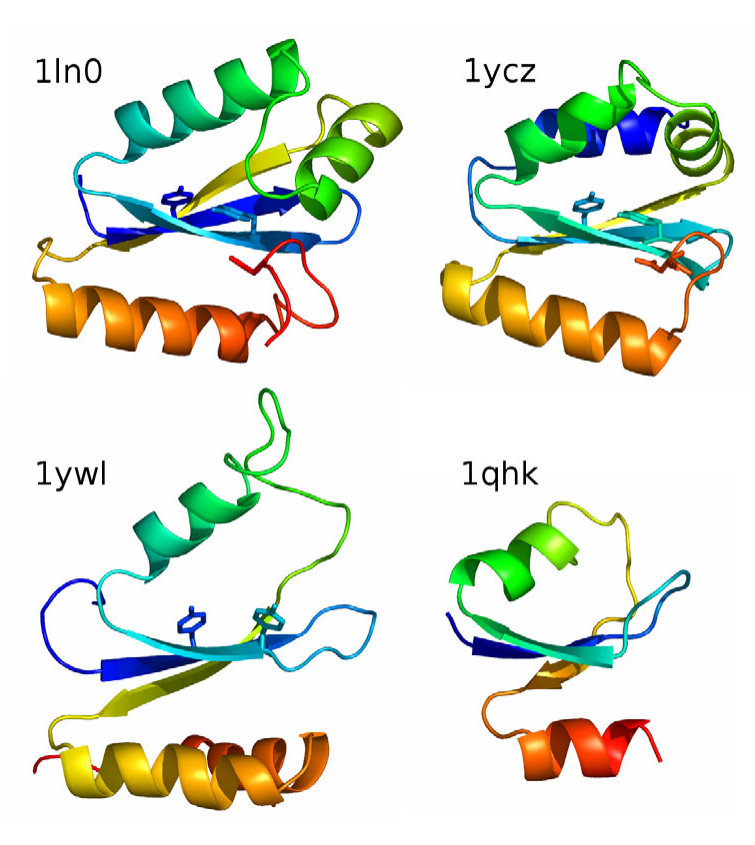
**Comparision between three-dimensional organization of GIY-YIG domains. **Structures of I-TevI (1ln0), UvrC (1ycz), Slx-1 (1ywl) and a domain of RNase H1 from *Saccharomyces cerevisiae *(1qhk) are shown in the cartoon representation, colored as a rainbow from the N-terminus (blue) to the C-terminus (red). The characteristic conserved Tyr residues from the GIY-YIG motif and the C-terminal Asn residue conserved in the UvrC-like lineage are shown as sticks.

Thus far, only a few structures of GIY-YIG nucleases have been determined, precluding structure-based calculation of the tree for the whole superfamily. Nonetheless, the existing data allow for comparison between the UvrC/I-TevI and Slx1-like varieties. The three-dimensional structures of bacterial members of the Slx1 family solved by the Nuclear Magnetic Resonance (NMR) (1ywl, and 1zg2 in the PDB) reveal considerable divergence from the structures of UvrC (1yd0, 1ycz) and I-TevI (1lk0/1mk0). In particular, the C-terminus, which contains the Asn residue we propose to be specific for the UvrC+ lineage, exhibits different conformations in Slx1 compared to UvrC and I-TevI (Figure 8). This result supports our sequence-based prediction that HEases and UvrC nucleases belong to a single lineage and are paralogous to Slx1. In this context it is noteworthy that the prokaryotic members of the Slx1 family typically lack any auxiliary domains and in general represent the minimal variant of the GIY-YIG domain. Therefore, we propose that the "standalone" Slx1-like domains represent the ancestral form of the GIY-YIG nuclease, from which the paralogous UvrC+ copy was derived and C-terminally extended to include the Asn residue (Figure 7). The structural similarity of the GIY-YIG fold to proteins such as the ribosomal protein L9 or the N-terminal domain of RNase HI [7] suggests that the ancestral GIY-YIG nuclease could have evolved from an ancient generic nucleic acid-binding (perhaps RNA-binding) domain. This scenario is similar to the putative origin of the LAGLIDADG fold, believed to have evolved from another nucleic acid-binding domain [57,58].

We hope that the results of our analyses described in this work will help to elucidate the function of the so far uncharacterized members of COG1833 and COG3680. They may be involved in NER in Archaea and Eukaryota, like their postulated Bacterial orthologs from the UvrC/Cho family, and they may exhibit different substrate specificities, in analogy to the functional divergence between UvrC and Cho. Alternatively, they could have been recruited to completely different processes. It is unlikely that COG3680 is essential for NER in Eukaryota, in which the major nucleases (XPG/Rad2 and ERCC1-XPF/Rad10-Rad1 for the 3' and 5' incision, respectively) have been determined (review: [59]). However, they could be involved in some specialized pathway of repair, e.g. in a loose analogy to Cho, which functionally substitutes for the N-terminal domain UvrC in making the 3' incisions near bulky lesions that are poor targets for UvrC. It will be interesting to study the role of COG1833 members in Archaea, both in species that also possess the "Bacteria-like" UvrABC system (such as Halobacteriales and Methanosarcinales) and in those, which lack this system (such as *M. jannaschii*). It is noteworthy that none of the fully sequenced Archaeal genomes contain UvrA or UvrB in the absence of UvrC, suggesting that members of COG1833, if involved in NER, must have different partners. On the other hand, the fusion of COG1833 with Exonuclease III in *M. jannaschii *suggests that these proteins may be involved in BER and that this pathway could be responsible for the repair of damages normally removed by NER in Bacteria and Eukaryota and those Archaea, which possess the classical UvrABC system.

The functional hypotheses listed above are based on the criterion of "guilt by association" [60], which states that domain fusions are often indicative of functional links. In addition to the experimentally verified functional connections, e.g. in the UvrC nuclease and in the Penelope elements, the recurring fusions with the RFM MTase and at least some fusions with the domains commonly involved in DNA repair (the most prevalent type of domains fused to the GIY-YIG nuclease) are significant and indicative of some functional associations. However, many fusions of the GIY-YIG domain with other domains may result from the extraordinary evolutionary mobility of the GIY-YIG domain and its ability to invade new loci rather than from the prefereed physical linkage with functional partners. In particular, the single instances of fusions of the GIY-YIG domain of the Slx1 family with the SPOUT MTase, as well as the unique insertion of the GIY-YIG domain into the HsdR subunit may not be necessarily functionally relevant. It will be interesting to determine if, in addition to the previously described selfish HEases and REases, other members of the GIY-YIG superfamily may function as molecular parasites.

It must be noted that throughout the evolution, the GIY-YIG domain was less successful than several other nuclease superfamilies in spreading to new loci, parasitizing different organisms, and adopting different functions. In particular, it was less successful than the PD-(D/E)XK domain in the formation of REases, which requires to recognize short sequences with very high specificity, and less successful than the LAGLIDADG domain in the formation of HEases that cleave within very long, degenerate sequences. This probably reflects the apparent poor potential of the GIY-YIG domain to form stable dimers or to develop peripheral elements that could be efficiently used either to form multiple contacts to the few recognized base pairs (as observed in most of REases from the PD-(D/E)XK superfamily) or to extend the protein-DNA interface to include contacts to multiple base pairs via the major grove (as in HEases from the LAGLIDADG superfamily). However, the GIY-YIG domain has been quite successful in formation of monomeric nucleases that utilize additional domains to recognize the DNA targets. This collection of domains can range from extremely simple DNA-binding elements (as in the case of I-TevI) to modules with independent enzymatic activities (as in the case of UvrC or the Penelope elements). With this respect, the GIY-YIG domain resembles the HNH domain, which is rarely found as a standalone nuclease and typically associates with different domains [61]. However, the HNH nuclease has been dramatically more successful than GIY-YIG both in formation of DNA structure-specific Holliday junction resolvases and recombinases, as well as DNA sequence-specific REases [62]. Thus, among the afore-mentioned nuclease superfamilies, GIY-YIG perhaps should be regarded as the least favorable template for the development of new functions.

### Summary

Our analysis reports identification of new members and extensive sequence analyses of the GIY-YIG superfamily. Based on the analysis of genomic distribution, patterns of domain fusions and phylogenetic considerations for individual families, we propose an evolutionary scenario that explains the emergence and development of the major branches of the GIY-YIG superfamily. All newly identified members are predicted to be nucleases and we suggest that most of them target DNA. The predictions reported in this article (in particular based on associations with protein domains with other functions) will facilitate the search for the possible substrates. The clustering of all members into well-defined subfamilies and detailed analysis of features characteristic for these subfamilies will help to select representatives for the experimental determination of function and structure and will facilitate the classification of novel members identified in the future. In particular, we propose to determine the possible function of members of COG1833 and COG3680 in the context of nucleotide or base excision repair, and to study the potential link of the bacterial members of COG3680 with pathogenicity.

## Methods

### Sequence database searches

A set of known members of the GIY-YIG superfamily (I-TevI GI: 6094464, UvrC from *Borrelia burgdorferi *GI: 2688360, Slx-1 from *Saccharomyces cerevisiae *GI: 6319705, ORF1 from the Penelope element of Drosophila virilis GI:15559194 were used as seeds in PSI-BLAST [63] searches of the non-redundant (nr) database and the publicly available complete and incomplete genome sequences at the NCBI [64]. For each sequence, the search was carried out in two versions: "conservative", with the expectation (e) value threshold for the retrieval of related sequences set to 10^-6 ^and the maximum number of iterations set to 6, and "aggressive", with the e-value threshold of 10^-2 ^and the maximum number of iterations set to 12. The final blast (blunt-end master-slave) alignments together with the predicted secondary structure were used to generate a set of query profile HMMs using HHmake from the HHsearch package [25]. The profile HHMs corresponding to all COG, KOG [65] and Pfam [66] entries were downloaded from the home site of HHsearch (the Department of Developmental Biology, MPI. Comparison of the profile HMMs (sequence+structure) was carried out using HHsearch [25], with default parameters. For the analysis of particular families, full-length sequences were retrieved and realigned using MUSCLE [26]. Manual adjustments were introduced into the multiple sequence alignment (MSA) based on the BLAST pairwise comparisons, secondary structure prediction, and results of the fold-recognition analyses (see below).

### Sequence clustering

To visualize pairwise similarities between and within protein families we used CLANS (CLuster ANalysis of Sequences), a Java utility that applies version of the Fruchterman-Reingold graph layout algorithm [27]. CLANS uses the P-values of high-scoring segment pairs (HSPs) obtained from an N × N BLAST search, to compute attractive and repulsive forces between each sequence pair in a user-defined dataset. The three-dimensional representation of sequence families is achieved by randomly seeding the sequences in space, and then moving them within this environment according to the force vectors resulting from all pairwise interactions, until convergence.

### Protein structure prediction

Secondary structure prediction and tertiary fold-recognition was carried out via the GeneSilico meta-server gateway [67]. Secondary structure was predicted using PSIPRED [68], PROFsec [69], PROF [70], SABLE [71], JNET [72], JUFO [73], and SAM-T02 [74]. Solvent accessibility for the individual residues was predicted with SABLE [71] and JPRED [72]. The fold-recognition analysis (attempt to match the query sequence to known protein structures) was carried out using FFAS03 [75], SAM-T02 [74], 3DPSSM [76], BIOINBGU [77], FUGUE [78], mGenThreader [79], and SPARKS [80]. Fold-recognition alignments reported by these methods were compared, evaluated, and ranked by the Pcons server [81]. The ultimate validation of fold-recognition results was accomplished by evaluation of the resulting three-dimensional models. Briefly, fold-recognition alignments to the structures of selected templates were used as a starting point for homology modeling using the "FRankenstein's Monster" approach [82], comprising cycles of model building, evaluation, realignment in poorly scored regions and merging of best scoring fragments. The final models were evaluated and analyzed using COLRADO3D [83]. The modeling protocol was essentially identical to that published in [84].

## Abbreviations

aa, amino acid(s); bp, base pair(s); nt, nucleotide; e, expectation; BER: base excision repair; NER: nucleotide excision ropair REase, restriction endonuclease; ORF, product of an open reading frame; GI, NCBI identification number;

## Authors' contributions

SDH carried out sequence analyses, clustering, participated in writing the manuscript prepared the figures. MF participated in profile-profile comparisons and genomic neighborhood analysis. JMB participated in all analyses, interpreted the data and drafted the manuscript. All authors have read and accepted the final version of the manuscript.

## Supplementary Material

Additional file 1 **All sequences of GIY-YIG superfamily members analyzed in this work. **Unaligned full-length amino acid sequences in the FASTA format. Names include: the species' name abbreviated with the first three letters of the genus and first three letters of the species (e.g. *Homo sapiens *= Homsap) and the NCBI Gene Identification number. Click here for file
